# Analysis of the impact of *DGAT1* p.M435L and p.K232A variants on pre-mRNA splicing in a full-length gene assay

**DOI:** 10.1038/s41598-023-36142-z

**Published:** 2023-06-02

**Authors:** Nicolas Gaiani, Lorraine Bourgeois-Brunel, Dominique Rocha, Arnaud Boulling

**Affiliations:** Université Paris-Saclay, INRAE, AgroParisTech, GABI, 78350 Jouy-en-Josas, France

**Keywords:** Gene regulation, RNA splicing, Agricultural genetics, Mutagenesis

## Abstract

*DGAT1* is playing a major role in fat metabolism and triacylglyceride synthesis. Only two *DGAT1* loss-of-function variants altering milk production traits in cattle have been reported to date, namely p.M435L and p.K232A. The p.M435L variant is a rare alteration and has been associated with skipping of exon 16 which results in a non-functional truncated protein, and the p.K232A-containing haplotype has been associated with modifications of the splicing rate of several *DGAT1* introns. In particular, the direct causality of the p.K232A variant in decreasing the splicing rate of the intron 7 junction was validated using a minigene assay in MAC-T cells. As both these *DGAT1* variants were shown to be spliceogenic, we developed a full-length gene assay (FLGA) to re-analyse p.M435L and p.K232A variants in HEK293T and MAC-T cells. Qualitative RT-PCR analysis of cells transfected with the full-length *DGAT1* expression construct carrying the p.M435L variant highlighted complete skipping of exon 16. The same analysis performed using the construct carrying the p.K232A variant showed moderate differences compared to the wild-type construct, suggesting a possible effect of this variant on the splicing of intron 7. Finally, quantitative RT-PCR analyses of cells transfected with the p.K232A-carrying construct did not show any significant modification on the splicing rate of introns 1, 2 and 7. In conclusion, the *DGAT1* FLGA confirmed the p.M435L impact previously observed *in vivo*, but invalidated the hypothesis whereby the p.K232A variant strongly decreased the splicing rate of intron 7.

## Introduction

The diaglyceride O-acyltransferase 1 encoded by the *DGAT1* gene is the enzyme catalysing the synthesis of triglycerides from diglycerides and acyl-coenzyme A. In 2002, the *DGAT1* p.K232A missense variant (BTA14:611019AA>GC) was associated with major variations of milk yield and composition in cattle^1^. The frequency of this variant depends of on the considered breed, but it is commonly found in the main breeds of dairy cows. Due to its important economical outcome on dairy industry, the role of the bovine *DGAT1* p.K232A variant has been extensively studied and reviewed^2^. In a global manner, the p.K232A variant was associated to lower fat yield, fat content, and protein content and higher milk production protein and lactose yield. From a functional point of view, this variant was shown to significantly decrease DGAT1 enzyme activity, in a concordant way with the diminution of milk fat percentage measured in heterozygous and homozygous p.K232A carriers^3^. Besides that, the use of a cryptic 5’ splicing site (5’SS) within the exon 8 was also detected, more markedly in animals harbouring the p.K232 allele. However, this specific mechanism was not identified as a major element that could explain changes in milk phenotypes, as the difference in terms of abnormal/normal transcript ratio between homozygous for p.K232 allele, homozygous for p.A232 allele, and heterozygotes animals was weak^3^.

Very recently, Fink *et al*. investigated the spliceogenicity of the p.K232A variant more deeply using an approach relying on complementary *in vivo* and *in vitro* methods^4^. After performing an association analysis using a mammary RNA-seq dataset representing 375 lactating cows and 3128 previously-imputed sequence variants lying in a 1Mbp interval overlapping the *DGAT1* gene, they observed that the p.K232A-containing haplotype associated *in vivo* with decreased *DGAT1* expression and modifications of splicing rate of several introns. Interestingly, the p.K232A variant was the top associated SNP regarding the modification of introns 2 and 7 splicing. Also, a minigene assay in bovine mammary epithelial (MAC-T) cells revealed that this variant decreased the splicing rate of intron 7, which means that the p.K232A causes the splicing modification of intron 7 observed *in vivo*.

From the Fink *et al.* study, the alteration of intron 7 splicing rate seemed to be the most convincing splicing effect directly attributed to the p.K232A variant, on the basis of both *in vivo* and *in vitro* evidences. Additionally, the authors pointed out a possible effect of this variant on the modification of the splicing rate of intron 2, as it was strongly associated with this latter *in vivo*, but were unable to validate this hypothesis because the minigene construct they developed was lacking *DGAT1* introns 1 and 2. They concluded that the alteration of *DGAT1* transcript expression could contribute to the phenotypic effects associated with the p.K232A variant.

The p.M435L variant is a rare alteration discovered by Lehnert *et al.* several years ago by screening lactation records of 2.5 million cows in order to identify significant deviation from means of milk protein and fat contents^5^. They filtered out 29 animals with abnormal milk parameters including one cow showing very low fat content, in which they subsequently identified the above-mentioned variant by genetic mapping and sequencing analysis. The detailed composition of milk samples collected from cows carrying this variant also revealed a substantial improvement in the ratio of saturated/unsaturated milk fat. In a second time, the authors performed qualitative and quantitative RT-PCR using total RNA extracted from liver of wild-type (WT) cattle in one hand, or alternatively from animals carrying the p.M435L variant at the heterozygous or homozygous state in the other hand. These RT-PCR analyses showed that this variant resulted in a nearly complete skipping of *DGAT1* exon 16. The resulting protein was expressed in *Saccharomyces cerevisiae* and was unable to transfer oleic acid from coenzyme A to diacylglycerol. By contrast, the WT protein expressed in same conditions is able to catalyse this reaction step needed to obtain a normal enzymatic product. This last observation supported the causative role of p.M435L in yielding the abnormal milk phenotype reported in this study.

Full-length gene assays (FLGA) have been routinely used to analyse numerous splicing variants within the human *SPINK1* gene^6–10^ and 5’SS GT>GC or GC>GT variants in 43 different genes^11,12^. In order to assess the spliceogenicity of a variant, the minigene assay is easier to employ than the FLGA and therefore more commonly used. The FLGA although presents two major advantages. First, the analysis of a splicing variant carried by a pre-mRNA transcript which has been generated by an episomal expression vector is more biologically relevant when integrating the whole gene sequence, since the nature and the length of the sequence context is known to impact RNA structures and splicing processes^13–15^. Next, the FLGA allows the assessment of any kind of genetic variants on splicing including deep intronic ones. Therefore, we created a bovine *DGAT1* FLGA in order to validate the effect of both p.M435L and p.K232A on splicing in a robust experimental system.

## Results

### Splicing pattern of the endogenous DGAT1 gene in the bovine MAC-T cells

**Figure 1:**
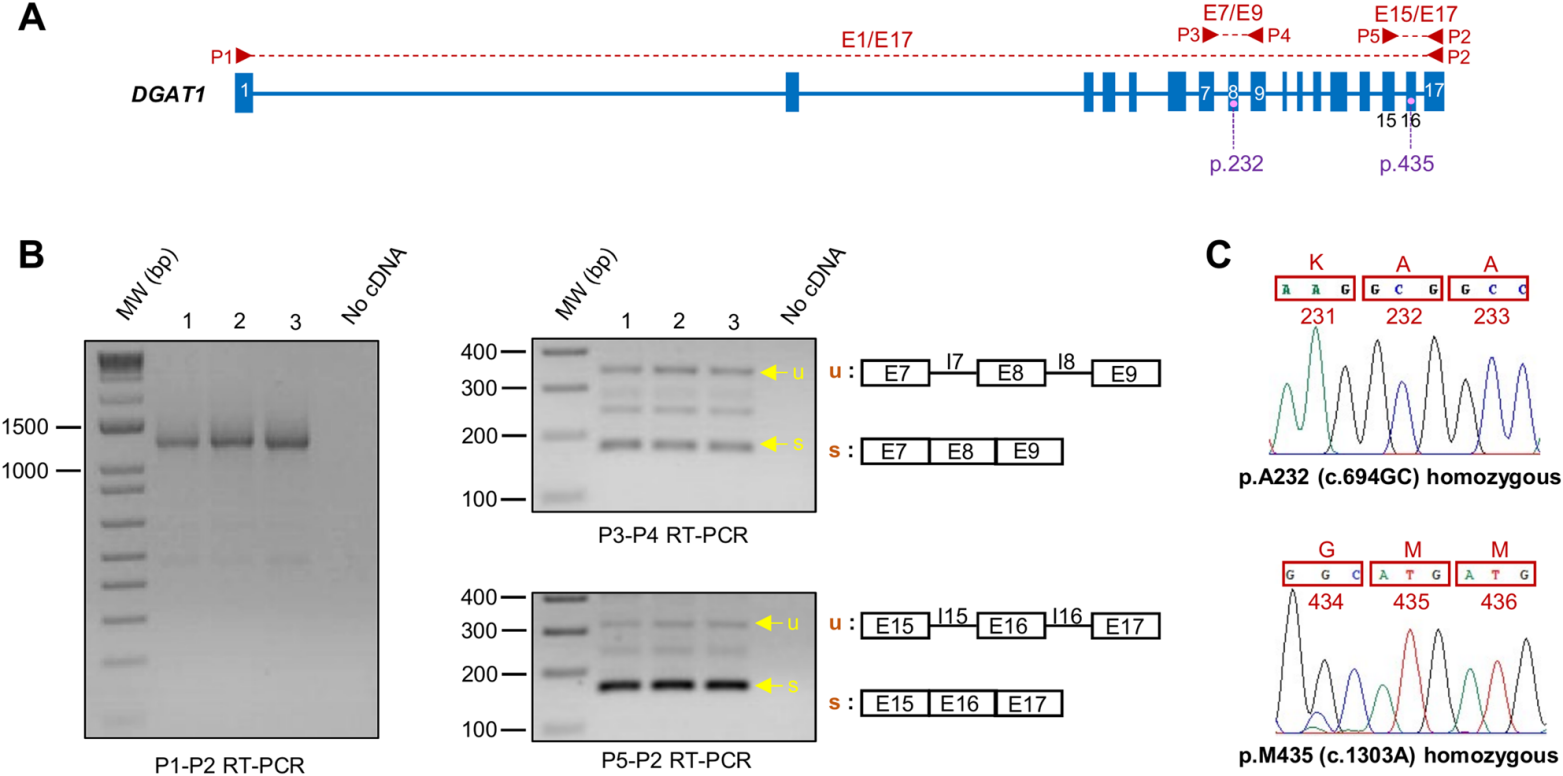
Qualitative RT-PCR analysis of the endogenous *DGAT1* splicing pattern in MAC-T cells. (**A**) Illustration depicting the structure of the bovine *DGAT1* gene. Blue boxes and lines represent bovine *DGAT1* exons and introns, respectively. Primers pairs P1-P2, P3-P4 and P5-P2 are indicated in red. (**B**) Gel electrophoresis of P1-P2, P3-P4 and P5-P2 RT-PCR products obtained from three different preparations of MAC-T cells. Spliced (s) and unspliced (u) products are indicated by yellow arrows and their structure is depicted on the right. **(C**) Genotype of MAC-T cells at positions p.232 and p.435.

The full-length *DGAT1* mRNA transcript from exon 1 to exon 17 was amplified from total RNA of three different preparations of non-transfected MAC-T cells using primer pair P1-P2 (Fig. 1A). A single amplicon whose size matches that of the spliced canonical transcript (*i.e.* 1220 bp) was observed by gel electrophoresis (Fig. 1B). In contrast, amplification of targeted regions of the transcript covering exons 7 to 9 and 15 to 17 using primer pairs P3-P4 and P5-P2, respectively, yielded multiple products (Fig. 1A, B; supplementary Figure 1). These have a size corresponding to the spliced *DGAT1* transcript, but also the unspliced form and in between partially spliced forms. At last, *DGAT1* genotyping of MAC-T cells showed that they were homozygous for p.A232 (c.694GC) and p.M435 (c.1303A) (Fig. 1C).

### Validation of the DGAT1 FLGA

**Figure 2:**
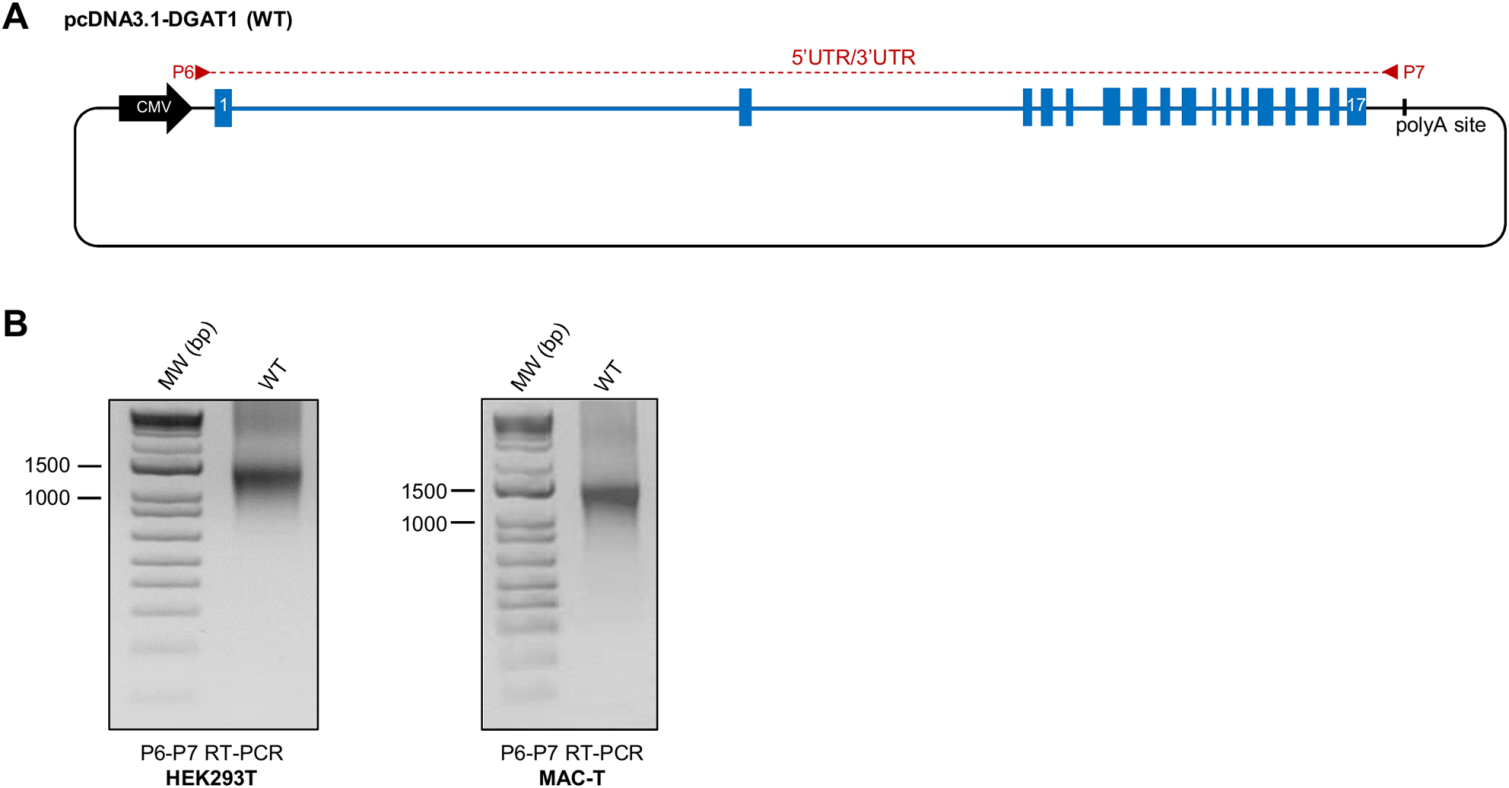
Validation of the *DGAT1* FLGA. (**A**) Illustration depicting the pcDNA3.1-DGAT1 (WT) construct. Blue boxes and lines represent bovine *DGAT1* exons and introns, respectively. Black line represents pcDNA3.1 (+) backbone. The P6-P7 primer pair and its target region is indicated in red. (**B**) Gel electrophoresis of P6-P7 RT-PCR products obtained from cells transfected with pcDNA3.1-DGAT1 (WT).

The whole genomic sequence of bovine *DGAT1* harbouring p.K232 and p.M435 alleles was successfully inserted in the pcDNA3.1 (+) expression vector to obtain the pcDNA3.1-DGAT1 (WT) construct (Fig. 2A), which was transfected in both MAC-T and human embryonic kidney (HEK293T) cell lines. The qualitative RT-PCR used to amplify the full *DGAT1* transcript invariably generated an amplicon at a size corresponding to the expected fully spliced *DGAT1* transcript in both cell lines (*i.e.* 1450 bp) (Fig. 2B). Of note, this product originated exclusively from the pcDNA3.1-*DGAT1* construct since 5’UTR and 3’UTR parts of the transcript targeted by primers P6 and P7 are related to the pcDNA3.1 (+) vector and not to the bovine genome, by contrast with primers P1 and P2 matching exon 1 and exon 17, respectively.

### The p.M435L variant causes DGAT1 exon 16 skipping in the human HEK293T cell line

**Figure 3:**
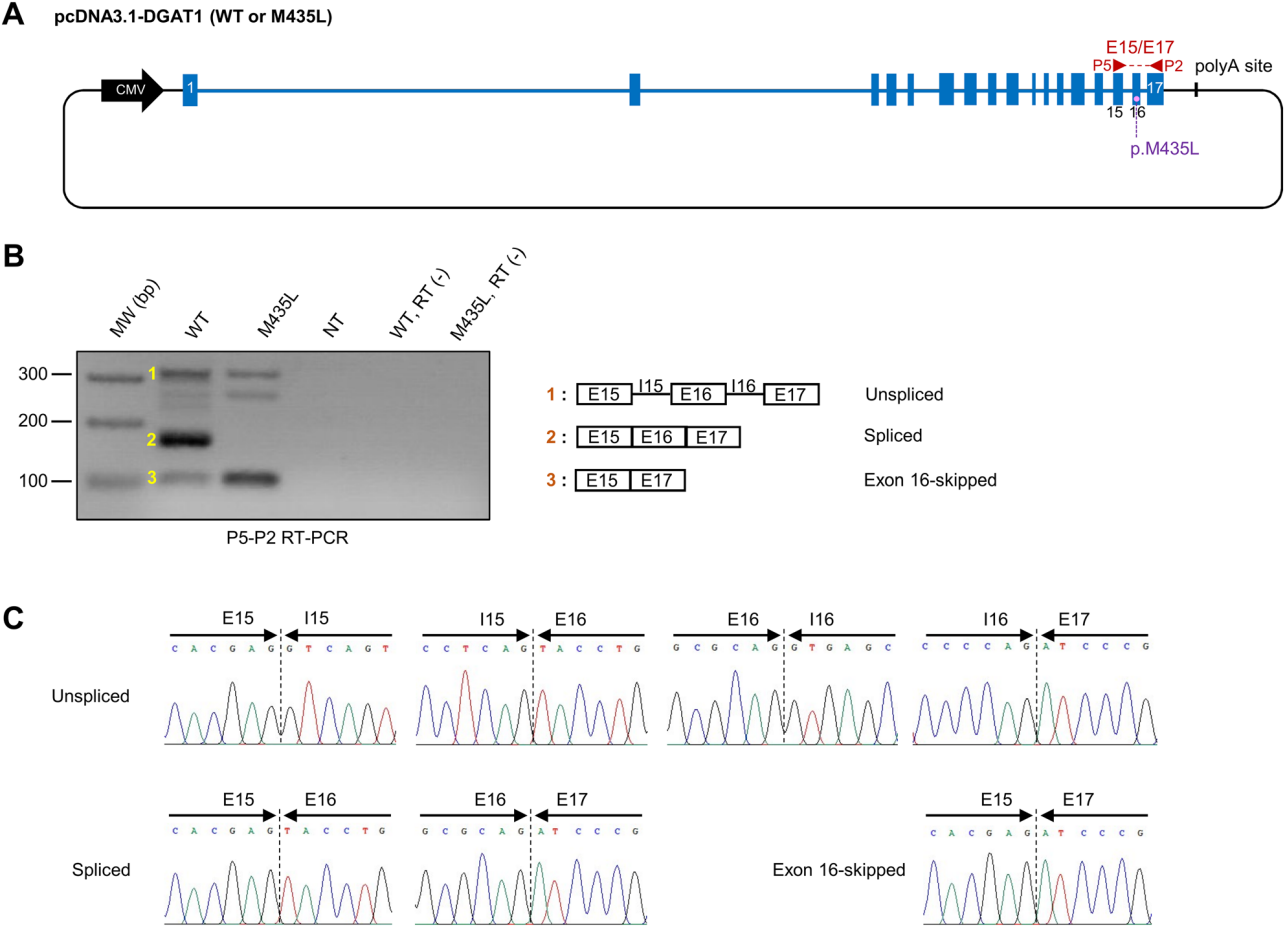
Qualitative RT-PCR analysis of HEK293T cells transfected with pcDNA3.1-DGAT1 (WT or M435L) constructs. (**A**) Illustration of plasmid constructs used for qualitative RT-PCR. pcDNA3.1-DGAT1 (WT) and pcDNA3.1-DGAT1 (M435L) are carrying alternatively the p.M435 or the p.L435 alleles, respectively. Blue boxes and lines represent bovine *DGAT1* exons and introns, respectively. Black line represents pcDNA3.1 (+) backbone. The P5-P2 primer pair and its target region is indicated in red. (**B**) Gel electrophoresis of P5-P2 RT-PCR products obtained from HEK293T cells transfected with pcDNA3.1-DGAT1 (WT or M435L). The structure of amplicons 1 to 3 is depicted on the right. NT, non-transfected ; RT (-), RT-PCR performed without Superscript III. (**C**) Validation of amplicon structure from (**B**) by Sanger sequencing. Only exon-intron and exon-exon boundaries are shown.

The effect of the p.M435L variant on exon 16 skipping was investigated by means of qualitative RT-PCR encompassing exons 15 to 17 and performed on cells transfected with both pcDNA3.1-DGAT1 (WT) and (M435L) constructs (Fig. 3A). The use of the human HEK293T cell line was preferred to those of the bovine MAC-T cell line. In fact, using a non-bovine cell line was the only way to avoid amplification of endogenous *DGAT1* transcripts that would have skewed the analysis, because PCR primers used to amplify *DGAT1* transcripts will synthesize exclusively transcripts generated by pcDNA3.1-DGAT1 constructs as they are bovine-specific.

The RT-PCR yielded multiple products (Fig. 3B, C; supplementary Fig. 2) which were purified, cloned into the pCR4-TOPO TA vector and sequenced. Three main PCR products corresponding to unspliced, spliced, or exon 16-skipped transcripts were identified. Exon 16-skipped transcripts were observed in the context of both (WT) and (M435L) constructs*.* By contrast, the product corresponding to correctly spliced transcript was exclusively observed for (WT) construct.

### The p.K232A variant slightly modifies the DGAT1 transcript pattern

**Figure 4:**
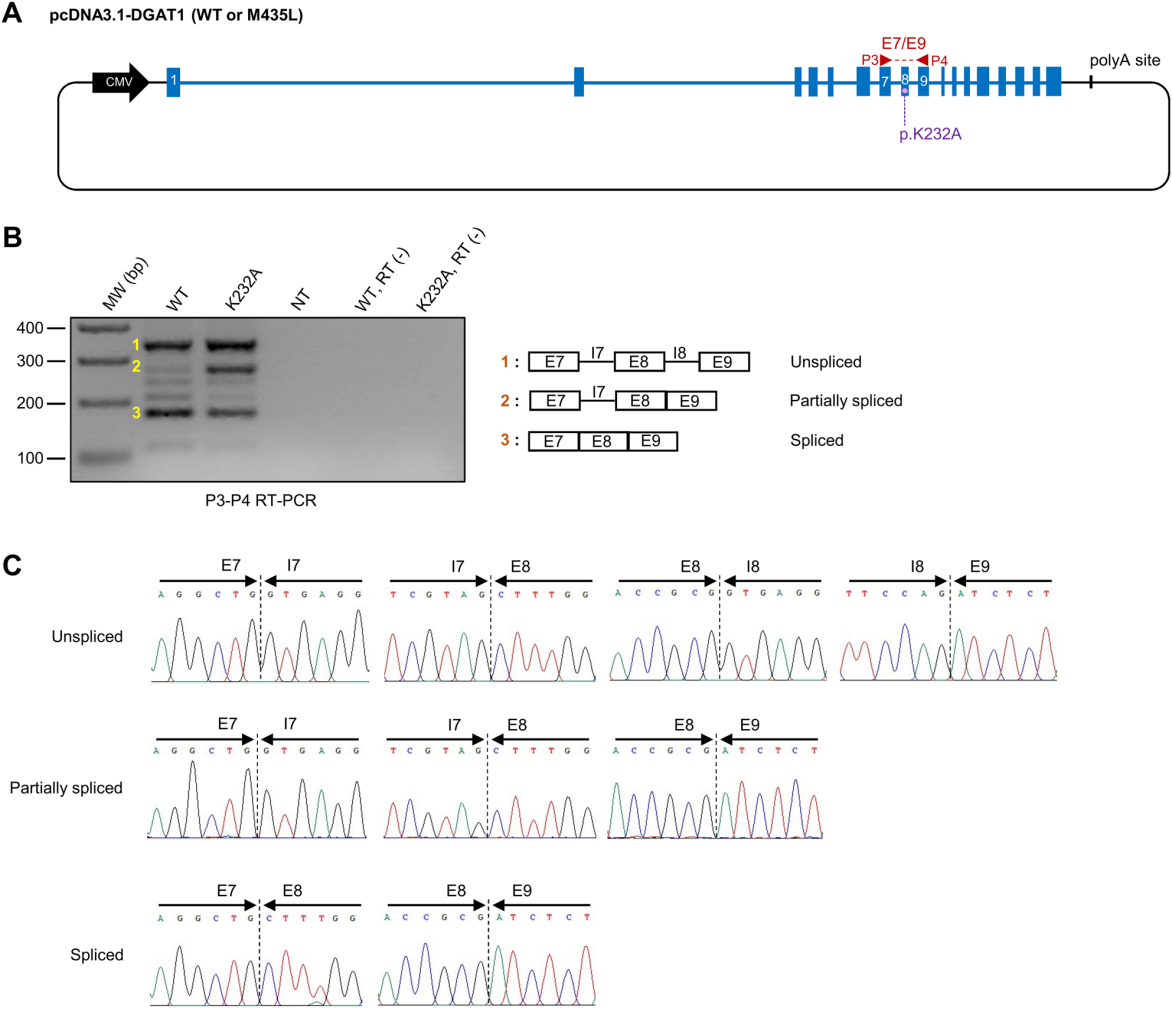
Qualitative RT-PCR analysis of HEK293T cells transfected with pcDNA3.1-DGAT1 (WT or K232A) constructs. (**A**) Illustration of plasmid constructs used for qualitative RT-PCR. pcDNA3.1-DGAT1 (WT) and pcDNA3.1-DGAT1 (K232A) are carrying alternatively the p.K232 or the p.A232 alleles, respectively. Blue boxes and lines represent bovine *DGAT1* exons and introns, respectively. Black line represents pcDNA3.1 (+) backbone. The P3-P4 primer pair and its target region is indicated in red. (**B**) Gel electrophoresis of P3-P4 RT-PCR products obtained from HEK293T cells transfected with pcDNA3.1-DGAT1 (WT or K232A). The structure of amplicons 1 to 3 is depicted on the right. NT, non-transfected ; RT (-), RT-PCR performed without Superscript III. (**C**) Validation of amplicon structure from (**B**) by Sanger sequencing. Only exon-intron and exon-exon boundaries are shown.

The qualitative effect of the p.K232A variant was investigated following the same approach as for the p.M435Lvariant (Fig. 4A). No difference was found in the nature of the observed transcripts, however one of the products seems to be present in greater quantity for the p.K232A variant (Fig. 4B, supplementary Fig. 3). It corresponds to an intermediate, partially spliced form of the transcript, in which intron 7 is still present, in addition to the 3 amplified exons (Fig. 4C). A very weak and diffuse band was barely visible at a molecular weight a little over 100 bp in both (WT) and (K232A) conditions. It could correspond to the minor alternative splicing isoform of *DGAT1* generated by the use of a cryptic splicing site within the exon 8 and described in previous studies^3,4^.

### The p.K232A variant does not significantly alter splicing rates of DGAT1 intron 1, 2 and 7 introns

**Figure 5:**
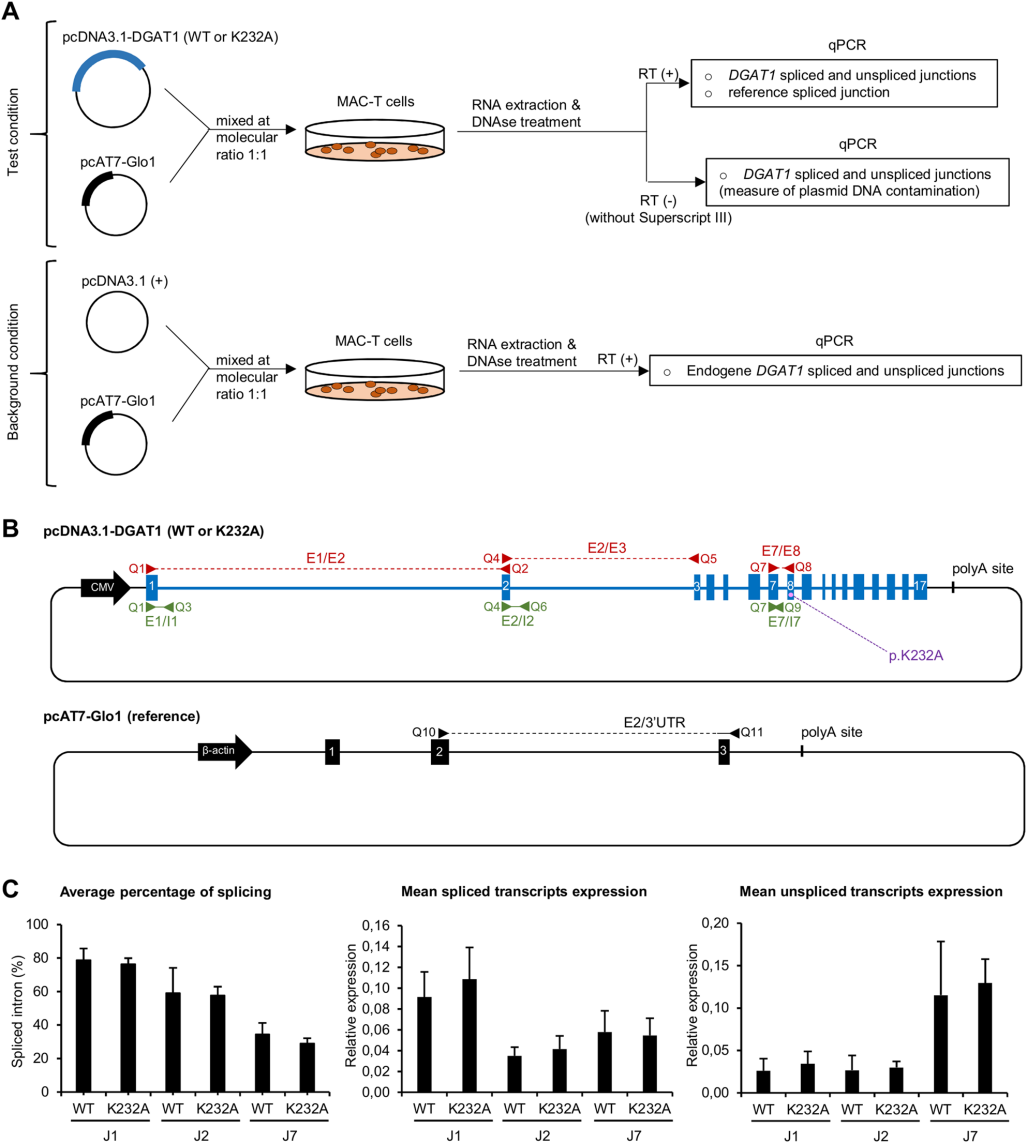
Quantitative RT-PCR analyses of MAC-T cells transfected with pcDNA3.1-DGAT1 (WT or K232A) constructs. (**A**) Experimental workflow of a quantitative RT-PCR experiment. (**B**) Illustration of plasmid constructs used for quantitative RT-PCR. pcDNA3.1-DGAT1 (WT) and pcDNA3.1-DGAT1 (K232A) are carrying alternatively the p.K232 or the p.A232 alleles, respectively. Blue boxes and lines represent bovine *DGAT1* exons and introns, respectively. Black line represents pcDNA3.1 (+) or pcAT7-Glo1 backbone. Primer pairs are indicated in red when targeting exon-exon boundaries and in green when targeting exon-intron boundaries. See materials and methods and Table 1 for more details.(**C**) Average percentage of splicing, mean spliced transcripts expression and mean unspliced transcripts expression for junction 1 (J1), junction 2 (J2) and junction 7 (J7) calculated from three independent transfection experiments performed in triplicate. Bars, SD. The relative expression attributed to *DGAT1* endogenous expression and DNA contamination was substracted from those obtained in test condition RT (+) to calculate the final relative expression presented here. No significant difference was observed between (WT) and (K232A) conditions in (**A**, **B** and **C**).

In the previous study from Fink *et al.*, the p.K232A variant was shown to markedly decrease splicing of intron 7 and was also suspected to alter the splicing of intron 2. For this reason, we designed quantitative RT-PCR to assess the relative expression of spliced and unspliced transcripts at junctions 2 and 7 (Fig. 5A, B). We also analysed junction 1 since, as junction 2, it was not included in the study by Fink *et al.* due to technical constraints. The MAC-T cell line was selected to perform the experiment, because quantitative RT-PCR enable to quantify and to remove *DGAT1* endogenous expression, by contrast with qualitative RT-PCR (Fig. 5A). pcDNA3.1-DGAT1 (WT) and (K232A) constructs were analysed in parallel to determine average percentage of splicing, mean spliced transcripts expression and mean unspliced transcripts expression for each junction 1, 2 and 7 (Fig. 5C). Differences were observed between junctions for these three parameters independently of the tested allele, illustrating that junctions 1, 2, and 7 presented different splicing efficiency. Contrarily, we did not observe any significant difference between (WT) and (K232A) conditions for any of these parameters when considering a given junction.

### Relative contribution of technical biases to DGAT1 relative expression

**Figure 6:**
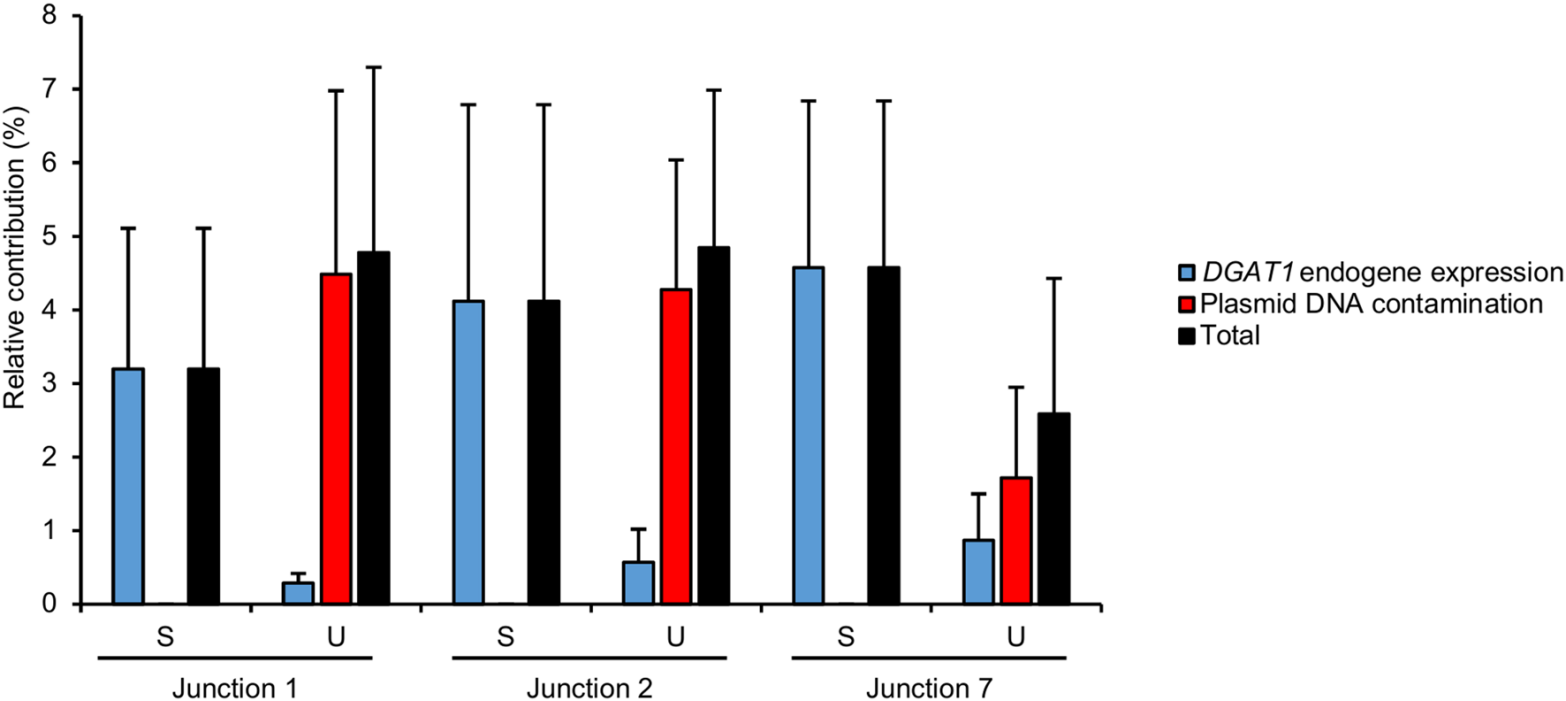
Mean relative contribution of *DGAT1* endogenous expression and plasmid DNA contamination to the relative expression measured by quantitative RT-PCR in the test condition RT (+) calculated from three independent transfection experiments performed in triplicate. Spliced junction (S), unspliced junction (U). Bars, SD.

In order to design the most reliable assay, we took into consideration two technical biases that may have decrease the accuracy of quantitative RT-PCR experiments that are i) the endogenous expression of *DGAT1*, and ii) the contamination of RNA samples by plasmid DNA. The mean relative contribution to the relative expression measured in the test condition of both these sources of error taken together was less than 5 %, regardless of the considered quantitative RT-PCR (Fig. 6). As explained in the material and methods section, the relative expression attributed to *DGAT1* endogenous expression and DNA contamination was substracted from those obtained in test condition RT (+) to get the corrected relative expression presented in Fig. 5C.

## Discussion

*DGAT1* is one of the most studied gene in bovine genetics due to its preponderant role in milk phenotypes. Although, only two loss-of-function variants of *DGAT1* have been described in cattle so far. The p.K232A and p.M435L variants have been associated with splicing defects *in vivo*^4,5^. However, their spliceogenicity has not yet been validated in a robust experimental system. We have developed a *DGAT1* FLGA to validate the effect of these variants on splicing, as to our knowledge, it is the most reliable system for this type of analysis. As p.M435L resulted in skipping of exon 16 *in vivo*, we used the FLGA in a qualitative way to analyse the sequence of the *DGAT1* transcript in the region encompassing exon 15 to exon 17. The *DGAT1* transcript generated from the pcDNA3.1-DGAT1 (WT) construct was carrying exon 16 and introducing the p.M435L variation into the construct led to complete skipping of this exon. In the *in vivo* study by Lehnert *et al.*, a very small amount of normally spliced transcripts was detected in cattle homozygous for p.M435L, which was a result comparable to what we have obtained in our system^5^. This validated the effect of the p.M435L variant and supported the use of the FLGA method to analyse other *DGAT1* splicing variants. Using the FLGA, we also observed the presence of the exon 16-skipped transcript in low quantity in the (WT) condition, which is not observed *in vivo*. This illustrates that although the FLGA method allows the study of a variant in the context of the complete transcript sequence, differences may persist between the splicing patterns of the endogenous transcript and the transcript obtained using FLGA. More generally, if a FLGA system does not generate the expected transcript or generates unexpected transcripts in too large quantities, we recommend not using it.

The subsequent qualitative analysis of the p.K232A variant revealed a slightly different splicing pattern for this variant compared to the WT condition. A partially spliced form of the transcript with retention of intron 7 was observed in greater quantity for the p.K232A variant. This first observation was in agreement with the hypothesis of Fink *et al*. that the p.K232A variant decreases the splicing rate of junction 7, however, no difference was observed between the WT and p.K232A conditions regarding the normally spliced product containing exons 7 to 9. This last point was in contradiction with the results of Fink *et al*. On the other hand, we have to keep in mind that the qualitative RT-PCR does not allow to quantify with precision the amplified products, it was then necessary to develop a quantitative RT-PCR to solve this issue, as performed by Fink *et al*.

Thus, we attempted to replicate the most compelling result from the Fink *et al*. study which was the decrease in the splicing rate of junction 7 caused by the p.K232A variant^4^. The possible effects on the splicing of introns 1 and 2 were also tested. In a manner comparable to theirs, we have set up quantitative RT-PCR and we have measured *DGAT1* spliced and unspliced transcripts relative expression at each junction, as the average percentage of splicing, in MAC-T cells transfected with pcDNA3.1-DGAT1 constructs. We did not observe any significant difference between (WT) and (K232A) conditions on any splicing parameters related to junction 1, 2 or 7. Conversely, the minigene assay developed by Fink *et al.* showed a 4-fold increase in terms of spliced transcript relative expression for the p.K232 allele by comparison to the p.A232 allele in the context of the junction 7, what resulted in a dramatic rise of intron 7 splicing ratio. Such a strong discordance may seem a little surprising but it can be explained by differences in terms of methodological choices or technical considerations between both studies. First, and as we have mentioned in the introduction, using a truncated or chimeric genomic sequence of a gene to perform episomal splicing reporter assays is less reliable than using the full gene sequence. A minigene harbouring all exons of a gene but lacking several of its introns as used by Fink *et al*. may generate strong false positives. This kind of bias has been unmasked by Wu *et al.*, who used FLGA to analyse the human *SPINK1* c.194G>A variant previously classified as strongly spliceogenic using minigene assay^6,16^. They revealed that this variant actually had no effect on splicing. Subsequently, it is also recognized that cell line-based experiments may suffer from a lack of reproducibility, especially when performed by different experimenters, in different laboratories, and using different cell preparations^17^. This may have contributed to conflicting results between our study and that of Fink *et al*.

We concluded that this variant had no substantial impact on the splicing of *DGAT1* junction 1, 2 and 7 by itself. Otherwise, our results are consistent with the fact that the variations in terms of expression and splicing efficiency observed *in vivo* are very weak, of the order of a few percent^4^. Such small variations may not be detected by quantative RT-PCR. We believe that the p.K232A variant probably has a small effect on splicing by itself, which is supported both by the fact that it is located in an exonic splicing enhancer^4^ and that we observed a possible effect by qualitative RT-PCR analysis, but which remains relatively anecdotal in comparison to its effect on the protein function. As suggested above, the very strong effect of this variant on junction 7 splicing as observed by Fink *et al.* in their minigene system was probably an artifact related to non-optimal methodological choices.

The last question addressed in our work concerned the relevance of verifying the impact of certain technical biases when using minigene assays or FLGA. Depending of the RT-PCR design, the specific quantitation of mRNA produced by an expression plasmid can be an issue due to i) confusion between endogenous and episomal expression of the target gene, and ii) contamination of total RNA by plasmid DNA^18^. The interference between endogenous and episomal expressions can be avoided by two ways. First, by using species-specific primers to exclusively amplify the target from plasmid construct, in combination with a cell-line from a different but genetically closely-related species as we did for p.M435L. Alternatively by using a cell-line in which the target gene is not expressed at all. The employment of a cell line originating from a tissue or a species different from those of the target gene is not really a matter since the splicing code is highly conserved between closely-related species^19^, as well as cell lines isolated from different organs showed most of the time comparable results when used to perform splicing reporter assays^20^. For instance, the HEK293T cells have been successfully used to analyse hundreds of variants in tens of genes expressed in many different tissues using minigene and midigene assays or FLGA (for examples, see references cited in introduction or^21–23^). Also, we showed that plasmid contamination can be decreased to an acceptable rate by using DNase treatment. Another option to avoid any amplification of contaminating plasmid DNA is to design RT-PCR primers only on exons separated by enough long introns, but this is not always possible^24^. Under the experimental conditions we used to perform quantitative RT-PCR in MAC-T cells, endogenous expression and plasmid contamination taken together counted for less than 5% of the *DGAT1* relative expression in average. Such contamination can be considered as low and does not represent a major source of error. We could have ignored it when calculating the *DGAT1* relative expression without it significantly changing the results. Nevertheless, we think everyone should be aware of this before starting a minigene or FLGA assay, and quantify this level of contamination if the RT-PCR design is likely to allow amplification of endogenous transcripts or contaminating plasmids. Indeed, if these contaminations are high, it can alter the reliability of the results. Finally, it is important to specify that plasmid contamination is a problem that must also be considered when performing qualitative RT-PCR. For such RT-PCRs it is not possible to quantify the contamination and substract it from the signal of interest, as we have done for quantitative RT-PCRs. It is therefore necessary to make an extra effort to eliminate all traces of plasmid DNA contamination in the RNAs, for instance by doing an additional step of DNAse treatment.

As closing remarks, we encourage the use of the FLGA method to analyse spliceogenic variants as it is the most robust episomal splicing reporter assay, especially to analyse hypomorphic splicing variants or variants involving complex mechanisms. Of course, minigene assay still remains a useful tool that fits well in many situations in combination with complementary functional, genetic and phenotypic data.

## Methods

### Construction of the pcDNA3.1-DGAT1 (WT) expression plasmid carrying full-length DGAT1 genomic sequence

A 8218-bp fragment containing the *DGAT1* genomic sequence from the end of first exon to the beginning of last exon (chr14: 604,331-612,548) was amplified from a bovine genomic DNA sample. It was obtained from a Holstein cow carrying the *DGAT1* p.K232A variant at the heterozygous state, which was previously whole genome sequenced^25^. The long range PCR was performed with 100 ng DNA in a 50 µL reaction mixture prepared on ice containing 25 µL 2X KAPA HiFi HotStart ReadyMix (Roche), and 0.3 μM each primer C1 and C2 (Table 1). The PCR program had an initial denaturation at 95 °C for 3 min, 32 cycles of denaturation at 98 °C for 20 s, annealing at 70 °C for 15 s, extension at 72 °C for 8 min 30 s, and a final extension step at 72 °C for 1 min. Amplicons were purified with the QIAquick PCR Purification kit (Qiagen). The pcDNA3.1 (+) expression plasmid was double digested with BamHI and XhoI before insertion of the previously generated amplicons by means of the In Fusion HD cloning Kit (Takara), according to the manufacturer’s instructions. Several constructs resulting from this process were analysed by Sanger sequencing to check the sequence at positions p.232 and p.435. A plasmid carrying p.K232 and p.M435 alleles was selected to be the pcDNA3.1-DGAT1 (WT) construct, and subsequently produced with QIAprep Spin Miniprep Kit (Qiagen) or the NucleoBond Xtra Midi EF (Masherey Nagel) and quantified using a NanoDrop™ One (Thermo Scientific). Finally, the construct was validated by doubled digestion with BamHI and XhoI and the sequence of the *DGAT1* gene inserted in the construct as well as that of the genomic DNA used for cloning were fully verified by Sanger sequencing. Two unwanted mutations were introduced into the construct during the PCR amplification step (*i.e.* BTA14:607550C>T, BTA14:608021C>T), but they were located outside the splice sites and far away from the two studied variants.

**Table 1:** Description of PCR primers.

Name	Sens	Sequence	Use
C1	F	TACCGAGCTCGGATCCACACAGACAAGGACGGAGAC	*DGAT1* cloning
C2	R	GCCCTCTAGACTCGAGCAAAGCAGTCCAACACCCACG	*DGAT1* cloning
M1	F	GGCCTTCACCGGCCTGATGGCGCAGGTGA	p.M435L mutagenesis
M2	R	TCACCTGCGCCATCAGGCGGTGAAGGCC	p.M435L mutagenesis
M3	F	GTAGCTTTGGCAGGTAAGGCGGCCAACGGGGGAGCTG	p.K232A mutagenesis
M4	R	CAGCTCCCCCGTTGGCCGCCTTACCTGCCAAAGCTAC	p.K232A mutagenesis
P1	F	ACACAGACAAGGACGGAGAC	Qualitative *DGAT1* RT-PCR
P2	R	GTAGTTGCCGCGGAAGAAG	Qualitative *DGAT1* RT-PCR
P3	F	TCAAGCTGTTCTCCTACCGG	Qualitative *DGAT1* RT-PCR
P4	R	GGGGCGAAGAGGAAGTAGTA	Qualitative *DGAT1* RT-PCR
P5	F	CAAGTGGGCAGCCAGGAC	Qualitative *DGAT1* RT-PCR
P6	F	CTGGCTAGCGTTTAAACTTAAGC	Qualitative pcDNA3.1 UTR RT-PCR
P7	R	GATCAGCGGGTTTAAACGGG	Qualitative pcDNA3.1 UTR RT-PCR
Q1	F	ACACAGACAAGGACGGAGAC	Quantitative *DGAT1* RT-PCR
Q2	R	GGGTCAAAGGTTAGGGGTCA	Quantitative *DGAT1* RT-PCR
Q3	R	TGAAGCCACTGTCAGAACTG	Quantitative *DGAT1* RT-PCR
Q4	F	AACTACCGTGGCATCCTGAA	Quantitative *DGAT1* RT-PCR
Q5	R	ACCGTGCGTTGCTTAAGAT	Quantitative *DGAT1* RT-PCR
Q6	R	ACAGAGCTCCATTCACCACA	Quantitative *DGAT1* RT-PCR
Q7	F	TCAAGCTGTTCTCCTACCGG	Quantitative *DGAT1* RT-PCR
Q8	R	CGAGGCAGCCCTCACCAG	Quantitative *DGAT1* RT-PCR
Q9	R	CTTACCTGCCAAAGCAGC	Quantitative *DGAT1* RT-PCR
Q10	F	ACGTGGATCCTGAGAACTTCA	Quantitative pcAT7-Glo1 RT-PCR
Q11	R	TAAACGGGCCCTCTAGAGC	Quantitative pcAT7-Glo1 RT-PCR

See Figs. 1A, 2A, 5B for details about “P” and “Q” primer pairs usage.

### Introduction of p.M435L and p.K232A variants into the pcDNA3.1-DGAT1 construct

*DGAT1* p.M435L (c.1303A>C) and p.K232A (c.694AA>GC) variants were introduced into the pcDNA3.1-DGAT1 (WT) construct by means of the QuikChange II XL Site‐Directed Mutagenesis Kit (Agilent Technologies). Mutagenesis was performed in a 51 μl mixture containing 2.5 U PfuUltra HF DNA polymerase, 1 μl dNTP mix, 5 μl 10× reaction buffer, 3 μl QuikSolution, 20 ng pcDNA3.1-DGAT1, and 125 ng each mutagenesis primer M1 and M2 or alternatively M3 and M4 (Table 1). The PCR program had an initial denaturation at 95 °C for 1 min, followed by 18 cycles of denaturation at 95 °C for 50 s, annealing at 60 °C for 50 s, and extension at 68 °C for 28 min, and a final extension at 68 °C for 7 min. The post-PCR reaction mixture was treated with DpnI at 37 °C for 1 hr then 4 µL of the resulting products were transformed into XL10‐Gold Ultracompetent cells (Agilent Technologies). Transformed cells were spread on LB agar plates with 50 μg/ml ampicillin then several selected colonies were used to prepare miniprep plasmid productions with QIAprep Spin Miniprep Kit (Qiagen). Miniprep plasmids were analysed by Sanger sequencing in order to verify the successful introduction of the desired substitution and one large scale NucleoBond Xtra Midi EF plasmid production was done and quantified for each variant. Constructs harbouring p.M435L and p.K232A variants were named pcDNA3.1-DGAT1 (M435L) and pcDNA3.1-DGAT1 (K232A), respectively.

### Cell culture and transfection, RNA extraction and reverse-transcription

HEK293T cells were cultured in the Dulbecco’s modified Eagle’s medium (DMEM) (Gibco) with 10% fetal calf serum (Sigma Aldrich). MAC-T cells were cultured in DMEM with 10% fetal calf serum supplemented with 4 mM L-Glutamine, 1% Penicilline/Streptomycine, 1 µg/L hydrocortisone and 50 mg/L insulin. Twenty-four hours before transfection, 3 × 10^5^ cells were seeded per well in six‐well plates. For qualitative reverse‐transcription polymerase chain reaction (RT‐PCR) analysis, 1 μg pcDNA3.1-DGAT1 (WT or M435L) plasmids extracted using QIAprep Spin Miniprep Kit, mixed with 3 μl Lipofectamine 2000 transfection reagent (Invitrogen), was used for transfection per well. For real‐time quantitative RT‐PCR analyses, pcDNA3.1-DGAT1 (WT or K232A) were mixed with pcAT7-Glo1 at equimolar ratio in order to reach a total amount of 1µg, then mixed with 3µL of Lipofectamine 2000 transfection reagent. Plasmids used for quantitative RT-PCR were all extracted using NucleoBond Xtra Midi EF. The pcAT7-Glo1 vector used in this study was provided by Scott I. Adamson and Brenton R. Graveley at the University of Connecticut, and was the modified version of the plasmid in which a splice acceptor site in the middle of intron 1 has been removed^20^. For the background control condition, pcDNA3.1-DGAT1 was replaced by pcDNA3.1 (+) empty plasmid using same molar quantities. Of note, pGL3-basic plasmid (Promega) was added to the mix to keep the final amount of 1 µg constant since pcDNA3.1 (+) had a lower molecular weight than pcDNA3.1-DGAT1. Forty‐eight hours after transfection, total RNA was extracted using the RNeasy Mini Kit (Qiagen) by adding an on-column DNase treatment. RNAs intended for qualitative RT-PCR subject to interference with plasmid contamination (*i.e.* RT-PCR P5-P2 and P3-P4 performed in the context of FLGA ; see below for a description of RT-PCR design) were submitted to an additional RNA Cleanup step using the RNeasy Mini Kit (Qiagen). RT was performed with the SuperScript III First-Strand Synthesis System for RT-PCR (Invitrogen) with 1 µg RNA, 2.5 µM Oligo(dT) 20, 500 µM each dNTP, 5 mM MgCl_2_, 5 mM dithiothreitol, 40 U RNaseOUT and 200 U Superscript III following the manufacturer’s instructions. Alternatively, only 100 ng RNA were used for qualitative RT-PCR P5-P2 and P3-P4 performed in the context of FLGA. Complementary DNA (cDNA) were degraded with 2 U RNaseH (Invitrogen). For each RNA sample, a negative control without SuperScript III enzyme was done to assess DNA contamination within total RNA samples.

### Qualitative RT-PCR analysis

Basically, qualitative RT-PCR was performed in a 50 µL reaction mixture containing 1.25 µL GoTaq® DNA Polymerase (Promega), 1.5 mM MgCl2, 200µM dNTPs, 0.5 µM primer pairs “P” as described in Table 1, and 2 µL cDNA. Note that 5 µL cDNA were used for qualitative RT-PCR P5-P2 and P3-P4 performed in the context of FLGA. Primers pairs P1-P2, P3-P4, P5-P2 and P6-P7 were used to amplify regions encompassing exons 1 to 17, exons 7 to 9, exons 15 to 17 and pcDNA3.1 5’UTR to 3’UTR, respectively. The PCR program had an initial denaturation at 95 °C for 2 min, followed by 30 cycles of denaturation at 95 °C for 30 s, annealing at 58 °C for 30 s, extension at 72 °C for 1 min 30 s, with a final extension step at 72 °C for 5 min. Main PCR products were gel purified and cloned into the pCR4-TOPO TA vector (Invitrogen) according to the manufacturer’s instructions. The sequence of clones corresponding to each transcript to be identified was then checked by Sanger sequencing using universal primers M13 forward and reverse.

### Quantitative RT-PCR analysis

Total RNA extracted from MAC-T cells co-transfected with both pcDNA3.1-DGAT1 and pcAT7-Glo1 were analysed by quantitative RT-PCR. pcAT7-Glo1 was used as the reference gene to calculate relative expression of spliced or unspliced junctions of transcripts originating from pcDNA3.1-DGAT1, in accordance with Pfaffl’s efficiency-corrected calculation model^26^. As described in Fig. 5B and in Table 1, *DGAT1* spliced transcripts expression were quantified using primer pairs spanning two consecutive exons, that are Q1 in exon 1 and Q2 in exon 2 for junction 1, Q4 in exon 2 and Q5 in exon 3 for junction 2, in addition to Q7 in exon 7 and Q8 in exon 7/8 for junction 7. *DGAT1* unspliced transcripts expression were quantified using primer pairs spanning one exon and the following intron, that is Q1 in exon 1 and Q3 in intron 1 for junction 1, Q4 in exon 2 and Q6 in intron 2 for junction 2, in addition to Q7 in exon 7 and Q9 in intron 7 for junction 7. To avoid experimental bias, the MAC-T *DGAT1* endogenous expression from cells transfected with empty pcDNA3.1 (+) was measured in parallel in each transfection experiment and substracted from those of pcDNA3.1-DGAT1 to eliminate the background signal (Fig. 5A). In addition, the RT-PCR negative control RT (-) was used to assess putative plasmid DNA contamination in each RNA sample used in the test condition, and to allow to substract it from the *DGAT1* relative expression measured in the RT (+) experiment of the test condition. It was obtained by performing the *DGAT1* RT-PCR without the Superscript III enzyme. After performing *DGAT1* PCRs on RT (-) samples, the calcul of the *DGAT1* relative expression generated by plasmid contamination in each RNA sample was done by expressing the *DGAT1* signal from RT (-) experiment in relation to the reference signal from the corresponding RT (+) experiment. Then, the resulting *DGAT1* relative expression representing the plasmid DNA contamination was substracted from those of the *DGAT1* RT-PCR assessing spliced and unspliced transcripts relative expression in RT (+) test conditions to get the corrected *DGAT1* relative expression presented in Fig. 5C. Finally, the average percentage of splicing observed at a given junction was obtained by dividing the mean spliced transcripts expression by the mean total transcripts expression. Three independent transfection experiments were analysed in triplicate for each condition, and the difference observed between pcDNA3.1-DGAT1 (WT) and (K232A) in each kind of analysis was assessed for significance by the Student’s t-test with threshold *p* < 0.05.

Technically, quantitative RT-PCR were performed in triplicate on a QuantStudio 12k Flex (Applied Biosystems). The reaction mixture contained 10 µL SYBR™ Master Mix PCR Power SYBR™ Green (Applied Biosystems), 0.6 µM primer pairs “Q” as described in Table 1 and Fig. 5B, and 5 µL of 40-fold diluted cDNA in a total volume of 20 µL. The PCR program had a first step at 50 °C for 2 min and a second step at 95 °C for 10 min, followed by 40 cycles of denaturation at 95 °C for 15 s, and annealing and extension at 60  °C for 1 min. It was subsequently followed by a melting curve program consisting of 95 °C for 15 s, 60 °C for 1 min and 95 °C for 15 s.

### Genotyping of MAC-T cells

A pellet containing 1.5 million MAC-T cells was used to perform DNA extraction using the Puregene cell & tissue kit (Qiagen). Genomic regions spanning exons 7 to 9 and 15 to 17 of *DGAT1* were amplified by PCR from this DNA using primer pairs P3-P4 and P5-P2, respectively, under the same conditions as described above in "Qualitative RT-PCR." The resulting amplicons were analyzed by Sanger sequencing using the same primers to determine the MAC-T genotype at positions p.232 and p.435 on the *DGAT1* gene.

## Supplementary Information

Supplementary Information.

## Data Availability

The authors confirm that the data supporting the findings of this study are available within the article.
