# Supplementary material for: Analysis of the impact of DGAT1 p.M435L and p.K232A variants on pre-mRNA splicing in a full-length gene assay
Source: Sci Rep. 2023 Jun 2;13:8999. doi: 10.1038/s41598-023-36142-z (PMC10238528; doi:10.1038/s41598-023-36142-z)
Supplement: Supplementary file 1 — Supplementary Information. [file 41598_2023_36142_MOESM1_ESM.pdf]

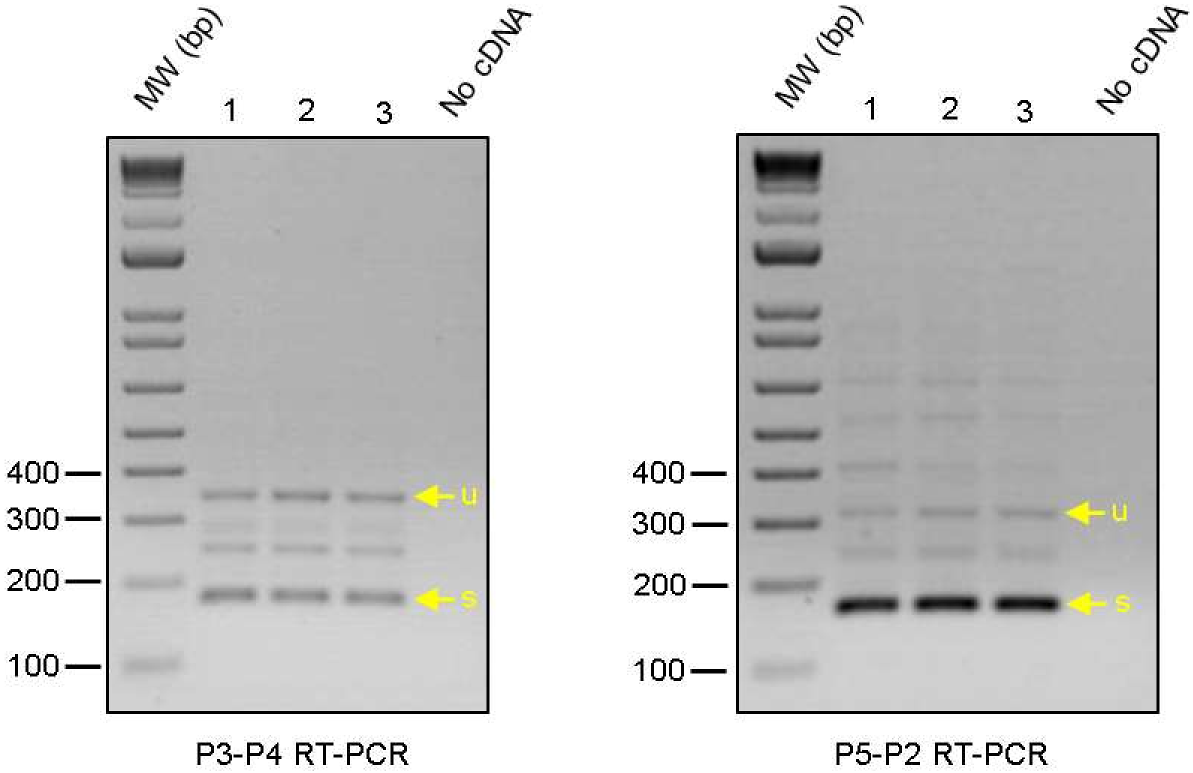

Supplementary Figure 1. Full picture of gel electrophoresis of P3-P4 and P5-P2 RT-PCRs products obtained from three different preparations of MAC-T cells. Spliced (s) and unspliced (u) products are indicated by yellow arrows. For the P5-P2 RT-PCR, faint bands corresponding to non-specific amplicons are visible above 400 bp.

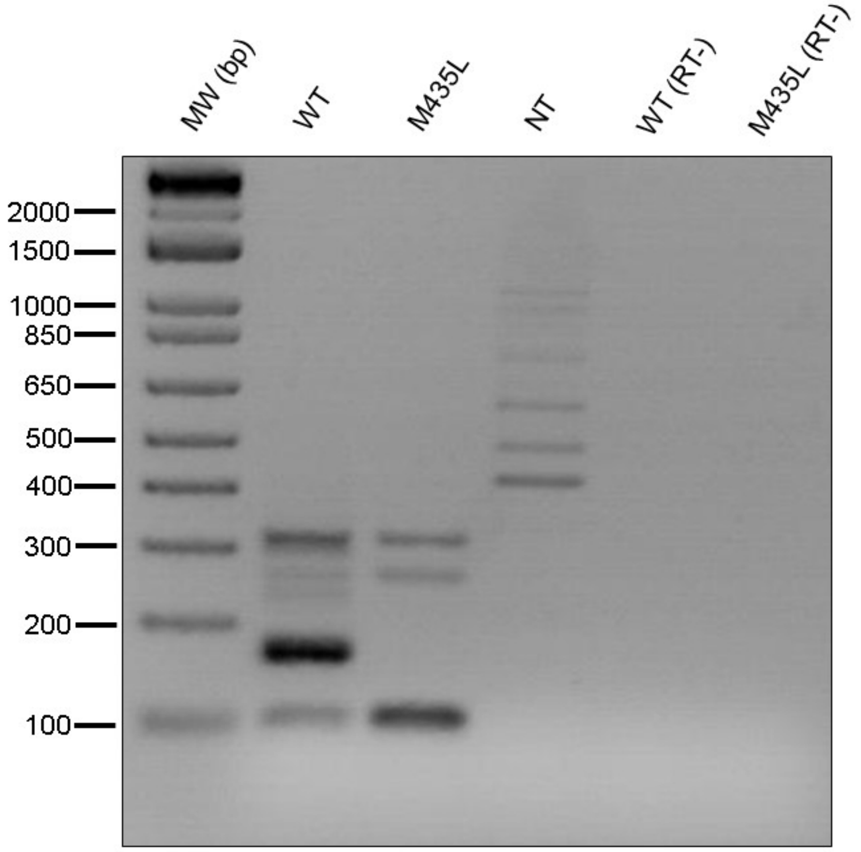

Supplementary Figure 2. Full picture of gel electrophoresis of P5-P2 RT-PCR products obtained from HEK293T cells transfected with pcDNA3.1-DGAT1 (WT or M435L). NT, non-transfected ; RT (-), RT-PCR performed without Superscript III. Unspecific products were observed in the NT condition. Of note, this does not have any consequence on the interpretation of WT and M435L conditions.

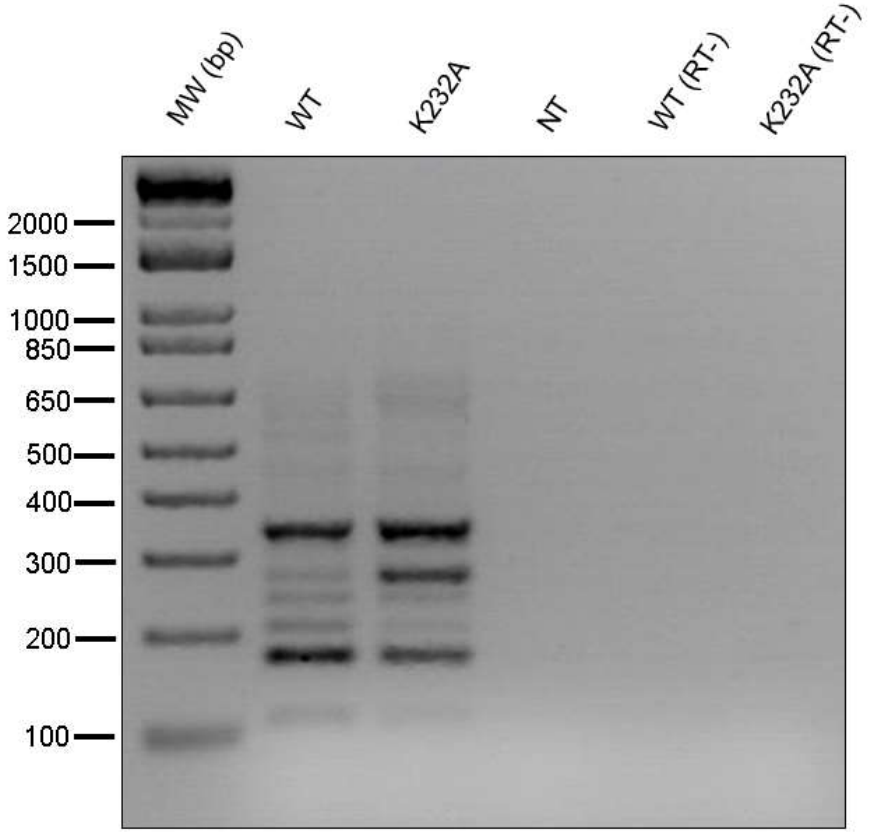

Supplementary Figure 3. Full picture of gel electrophoresis of P3-P4 RT-PCR products obtained from HEK293T cells transfected with pcDNA3.1-DGAT1 (WT or K232A). NT, non-transfected ; RT (-), RT-PCR performed without Superscript III.
